# Fabrication and In Vitro/In Vivo Appraisal of Metronidazole Intra-Gastric Buoyant Sustained-Release Tablets in Healthy Volunteers

**DOI:** 10.3390/pharmaceutics14040863

**Published:** 2022-04-14

**Authors:** Mohammed H. Elkomy, Heba A. Abou-Taleb, Hussein M. Eid, Heba A. Yassin

**Affiliations:** 1 Department of Pharmaceutics, College of Pharmacy, Jouf University, Sakaka 72341, Saudi Arabia; 2 Department of Pharmaceutics and Industrial Pharmacy, Faculty of Pharmacy, Merit University (MUE), Sohag 82755, Egypt; heba.ahmed@merit.edu.eg; 3 Department of Pharmaceutics and Industrial Pharmacy, Faculty of Pharmacy, Beni-Suef University, Beni-Suef 62511, Egypt; hussien.eid@pharm.bsu.edu.eg; 4 Department of Pharmaceutics, Faculty of Pharmacy, El Saleheya El Gadida University, El Saleheya El Gadida 44813, Egypt; drhobaa48@gmail.com

**Keywords:** floating tablets, drug delivery system, sustained-release system, HPLC assay of metronidazole, in vitro and in vivo evaluation of metronidazole

## Abstract

*Helicobacter pylori* is thought to be the most common cause of peptic and duodenal ulcers. Eradication of this organism is now considered one of the lines of treatment of gastric and duodenal ulcers. This can be achieved via local delivery of antibacterial agents in high concentrations. Accordingly, our objective was to fabricate and evaluate sustained release floating tablets for metronidazole to extend the gastric residence period and control the release rate of metronidazole. Floating tablets containing cellulose derivatives and Avicel were prepared using direct compression. The rate of metronidazole release from the floating tablets (K = 6.278 mg min^−1/2^) was significantly lower than that from conventional tablets (K = 10.666 mg min^−1/2^), indicating sustained drug release, according to the Higuchi model, for more than 6 h in an acidic medium of 0.1 N HCl. In vivo study in healthy volunteers revealed significantly improved bioavailability; increased Tmax, AUC, and MRT; and significantly lower absorption rate constant after a single oral dose of 150 mg metronidazole as floating tablets. In addition, the significant increase in MRT indicated an in vivo sustained drug release. The floating tablets provided several benefits, including ease of preparation, absence of effervescent ingredients, and reliance on a pH-independent gel-forming agent to deliver metronidazole in a sustained manner. In conclusion, the prepared tablets could be promising for enhancing both local and systemic metronidazole efficacy.

## 1. Introduction

*Helicobacter pylori* (*H. pylori*) is a Gram-negative, helically shaped, and microaerophilic bacterium usually found in the stomach [1]. Infection with *H. pylori* is asymptomatic and without complications in about 90% of infected individuals [2]. However, peptic ulcers may be experienced in 10% to 20% of the individuals infected with *H. pylori* [3,4]. Signs of acute infection that may be encountered involve acute gastritis with abdominal pain and stomachache or nausea [5]. The symptoms may progress to chronic gastritis with events of non-ulcerative dyspepsia, frequent gastric pain, bloating, loss of appetite, belching, and sometimes vomiting [6]. Stomach bleeds manifested as black stools may also occur with chronic gastritis. Continuous bleeding for an extended period may result in anemia, weakness and fatigue, hematochezia, hematemesis, and melena in the case of heavy bleeding.

People with *H. pylori* infection often show signs of inflammation of the pyloric antrum (the gate connecting the stomach to the duodenum), leading to duodenal ulcers. Another form of inflammation that could be experienced with *H. pylori* infection is the inflammation of the corpus (i.e., the body of the stomach), which most likely would lead to gastric ulcers [7,8]. The development of gastric or colorectal polyps in individuals infected with *H. pylori* may also occur [9]. Although these polyps usually pass unnoticed, sometimes gastric polyps may produce heartburn, dyspepsia, upper tract bleeding, and, less often, gastric outlet obstruction [10]. Likewise, colorectal polyps may produce some annoying symptoms, such as abdominal discomfort, weight loss, diarrhea, constipation, and even rectal bleeding and anemia [11]. The utmost malignant effect a chronic infection with *H. pylori* can do is increasing the risk of acquiring cancers along the alimentary canal [12].

**Figure 1:**
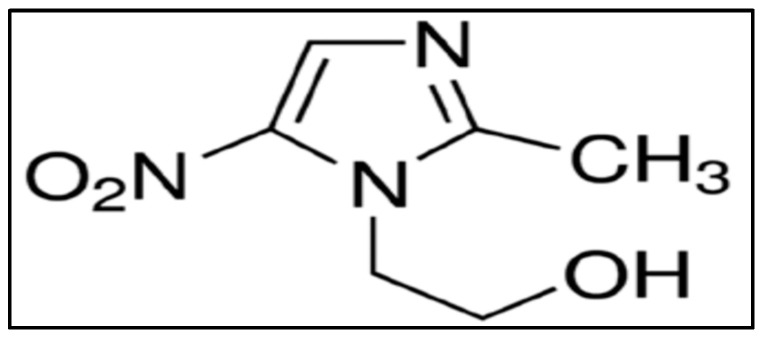
Chemical structure of metronidazole.

Metronidazole is 2-(2-methyl-5-nitro-1H-imidazol-1-yl) ethanol antimicrobial agent (Figure 1), which is capable of eradicating numerous anaerobic bacteria [13,14]. Metronidazole has been actively used as an adjunct agent to manage H. pylori infections affecting the upper gastrointestinal tract (GIT) [4]. Since the *H. pylori* infection site is located in the stomach’s mucus lining, treatment of upper GIT infections is extremely challenging [15]. An effective treatment would provide adequate amounts of antimicrobial agents to the upper tract for an extended period. This is probably why conventional formulations have proven ineffective when it comes to treating local gastric infections. The short gastric residence time and uncontrolled drug release of the conventional formulations hinder the achievement of adequate results. Frequent dosing is often required with this type of formulation to overcome the highly variable gastric emptying [16,17]. Gastric emptying interferes with the work of conventional formulations by transferring the formulation quickly to the intestine and interrupting the process of drug release to the mucus site of the upper GIT.

To enhance the bioavailability of drugs with a narrow absorption window from the upper GIT, gastro-retentive delivery systems, including floating drug delivery systems, are considered the system of choice due to their virtue of being retained in the stomach for a more extended period as compared with conventional drug delivery systems [18]. Floating systems are ideal for poorly absorbed medications in the lower intestine, drugs that are degraded in the lower GIT, pharmaceuticals that are absorbed mainly from the stomach and upper intestine, or drugs that act locally in the upper GIT. On the other hand, drugs that cause gastric lesions or degrade in the acidic medium are not suitable candidates for floating systems. Factors that moderate the gastric retention of floating drug delivery systems in the stomach include the stomach pH, dosage form size, food intake, and biological factors, such as gender, body mass index, age, posture, and disease status. Floating tablets, beads, granules, and microspheres of floating drug delivery systems have recently gained significant importance [19].

Therefore, in this work, we sought to develop and evaluate slow-release floating tablets for metronidazole to prolong metronidazole gastric resistance time while controlling for the release of the drug.

## 2. Materials and Methods

### 2.1. Materials

Metronidazole was procured from Sigma-Aldrich (St. Louis, MO, USA). Hydroxypropyl cellulose (HPC) and hydroxypropyl methylcellulose (HPMC, low-molecular-weight K30 (1–5% dry solution and 5–10% in tablet: 30,000 centipoises)) were purchased from Shinyo Pure Chemicals Co. (Osaka, Japan). Avicel and hydroxyl methylcellulose (HMC, viscosity of 2% aqueous solution at 25 °C: 4000 centipoises) were procured from Sd fine chemicals (Mumbai, India). Deionized water was purified using a Milli-Q system (Millipore, Modified., Burlington, MA, USA). All solvents used for HPLC analysis were HPLC grade. Other excipients were of analytical grade.

### 2.2. Methods

#### 2.2.1. Formulation of Metronidazole Floating Tablets

Optimization of the tablet formulation was achieved by studying the effects of different factors on the floating aspects of the tablet formulations. The factors comprised hydrogel composition, tablet weight, and drug content. All formulations were prepared via direct compression (Rotary Tablet Machine = stokes M 912-512 L, U.S.A.). All powders were passed through an 80-mesh screen prior to the preparation of the tablets. Powders were weighed and then mixed, employing geometric dilution. Magnesium stearate (1% w/w) was then added to the powder blend as a lubricant, and the final mixture was compacted on a rotatory tablet press using a 14 mm standard punch with a round flat face.

#### 2.2.2. Effect of Hydrogel Composition on Buoyancy

Different formulations were prepared in an attempt to maximize metronidazole content in the floating tablets. The tested polymeric hydrogels were HMC, HPC, and HPMC (Table 1).

#### 2.2.3. Effect of Tablet Weight on Buoyancy

The effect of tablet weight on buoyancy was studied by preparing different formulations containing HPMC and Avicel (1:1) and having different weights of 400, 500, 600, 700, and 800 mg.

#### 2.2.4. Effect of Hardness in Relation to Tablet Weight on Buoyancy

Each formulation was exposed to a spectrum of compression forces in order to obtain tablets with various hardness ranges, and samples of each hardness range were tested for buoyancy. The maximum hardness below which the tablets immediately floated and above which the tablets sunk was determined for each formulation. This parameter denoted the maximum hardness of immediate buoyancy (MHIB) and was determined for each formulation.

#### 2.2.5. Effect of Drug Content on Buoyancy

Different formulations were prepared in order to maximize metronidazole content in the floating tablets. All formulations contained HPMC as the main matrix former in which metronidazole was dispersed in different concentrations of 50, 100, 120, 150, and 200 mg/tablet to form homogenous mixtures. Each formulation was exposed to a spectrum of compression forces to yield tablets with variant hardness ranges and densities. For all formulations, tablet weight was maintained constant at 800 mg.

#### 2.2.6. Design of Metronidazole Tablets

The designed metronidazole tablets were optimized with respect to the hydrogel composition and tablet weight. HPMC was the selected hydrogel to represent the tablet matrix, and the chosen tablet weight was 800 mg. The tablets were prepared to have the lowest pharmaceutically accepted hardness range of 5–6 kg. The drug content in the designed formulations was varied from 0 to 250 mg. The formulations were denoted F1 to F7 (containing 0, 50, 100, 120, 150, 200, and 250 mg drug, respectively) and were subjected to in vitro evaluation. Conventional 800 mg tablets containing 150 mg metronidazole, Avicel, and magnesium stearate (1% w/w) were prepared via direct compression utilizing the same procedure described above. The conventional tablets were used as reference tablets with the same dose strength as the optimized sustained release floating tablets (150 mg metronidazole).

#### 2.2.7. In Vitro Evaluation of Proposed Tablets

Hardness, friability, content uniformity, weight variation, and thickness were investigated. For each formulation, the hardness of 10 tablets (Erweka, Type TB 24, Heusenstamm, Germany), friability of 10 tablets (Erweka, Type IAP, Heusenstamm, Germany), thickness of 6 tablets (vernier caliper), content uniformity of 10 tablets, and weight variation of 20 tablets were evaluated.

#### 2.2.8. Density Measurements

The formulated tablets were evaluated for apparent density, which was calculated by dividing the mean weight (800 mg) by the mean external volume. The tablet volume was calculated from the mean tablet thickness (h) and radius (r = 7.0 mm) utilizing the following equation:

$$\mathrm{Tablet}\;\mathrm{volume}=\pi \cdot r^{2}\cdot h$$

#### 2.2.9. Buoyancy Study

Buoyancy was investigated using a USP type 2 apparatus (Paddle type, Copley, England) with 0.1 N HCl (900 mL) as the dissolution medium, a temperature of 37 °C ± 0.5, and a paddle rotation speed of 100 rpm. For each run (*n* = 6), the time taken by an immersed tablet to rise to the surface of the dissolution medium was recorded as the floating lag time. Moreover, the time duration continuously spent by a tablet on the surface of the dissolution medium was recorded as the total floating time. The floating lag time and the total floating time make up the in vitro buoyancy characteristic of a given tablet.

#### 2.2.10. In Vitro Release of Metronidazole Floating Tablets

Metronidazole was dissolved in 100 mL of 0.1 N HCl to give a stock solution with a concentration of 1 mg/mL. Appropriate volumes of the stock solution were diluted by adding 0.1 N HCl to 50 mL to obtain different concentrations in the range of 8–20 µg/mL. A UV-visible recording spectrophotometer (SHIMADZU (UV-160A), Japan) was employed to determine the absorbance of each concentration at 277 nm [2]. A standard calibration curve was constructed by plotting absorbance values against concentrations.

The release of metronidazole from the prepared tablets was tested using a USP type 2 apparatus (Paddle type, Copley, England) with 0.1 N HCl (900 mL) as the dissolution medium, a temperature of 37 °C ± 0.5, and a paddle rotation speed of 100 rpm. Every 10 min for a continuous 6 h, samples from the dissolution medium were withdrawn and then replaced with fresh medium. Following filtration through a Millipore filter (0.45 µm), samples were suitably diluted and assayed for metronidazole content at 277 nm with the UV-visible spectrophotometer. Metronidazole concentrations were calculated from the constructed calibration curves, and floating characteristics were tested during the dissolution test. The optimized formula remained floating for more than 8 h. To assess the potential mechanism of metronidazole release from tested formulations, the release data obtained were fitted into three kinetic models, namely, zero order, first order, and Higuchi models [20].

## 3. Analysis of Metronidazole in Human Serum

### 3.1. HPLC of Metronidazole

The concentration of metronidazole was measured in serum samples via an HPLC system (Water Auto-Sampler, model 717 plus; solvent delivery, model 600; Tunable Absorbance Detector, model 486; and Millennium 2010 chromatography manager). Separation was achieved using a reversed-phase column (Waters Mondpak C18, 15 × 0.39 cm, 125A^o^10 µm). An aliquot (250 µL) of serum and 30% trichloroacetic acid (100 µL) were mixed, and the mixture was vortexed using a Vortex Mixer (Thermolyne corporation, Dubuque, IA, USA) for 30 s and then centrifuged for 25 min using a centrifuge (Minor 35, England) at 1800× *g*. A 50 µL sample of the clear supernatant was injected into the HPLC system. A reversed-phase column was employed for the separation process, and UV detection was monitored at 320 nm. The eluent flow rate was set to 2 mL/min. A concentration of 0.005 M monobasic potassium phosphate (pH 4), methanol, and acetonitrile (97:2:1) made up the mobile phase. Blank human serum samples were spiked with standard amounts of metronidazole to establish calibration curves. The peak area was measured to determine the metronidazole concentration in the assayed samples. The acquired data were digested and processed using Waters Millennium 2010 chromatography manager [21].

### 3.2. HPLC Assay Validation Procedure

The HPLC method was assessed in terms of linearity, selectivity, precision, and accuracy [22,23]. Linear calibration curves were established from peak areas, and the conforming metronidazole concentration ranged from 0.1 to 12 µg/mL in each calibration standard. The accuracy was defined as the absolute mean value of the ratio of the peak calculated mean values of unknown samples and their nominal values, expressed as a percentage. The measured concentrations of the within-day and between-day analysis were compared to the nominal concentrations to determine the accuracy of the assay. The precision of the assay was measured using the within-day and between-day percent coefficient of variation over the concentration range of 8–20 µg/mL over the course of the validation. The assay’s within-day accuracy and precision were determined by analyzing three replicates of the calibration curves on the same day. The between-day accuracy and precision were evaluated by analyzing six different calibration curves on six different days during the study period. The retention time of the drug in the standard and study samples were compared to define any endogenous peaks appearing at the same retention time for metronidazole. The retention time precision was measured using the run percent coefficient of variation (*n* = 6).

### 3.3. In Vivo Study

A single oral dose pharmacokinetic study was conducted on human volunteers to get insight into the impact of the sustained release buoyant tablets on the bioavailability of metronidazole compared to conventional tablets.

### 3.4. Volunteers

Six healthy adult male volunteers were recruited to participate in the conducted study (Table 2). The study was approved by the Ethical Committee of the Faculty of Pharmacy, Minia University (HV12/2020), and followed the Declaration of Helsinki. The nature of the study was explained to the participating subjects, and written consent was obtained. The average age was 31 years (range 29–34), and the average weight was 66 kg (range 60–72). Physical examinations showed that all volunteers had no clinical evidence of impaired renal, hepatic, cardiac, or pulmonary functions. Blood urea nitrogen and serum creatinine levels for all subjects were within the normal range, indicating normal kidney function. The intake of all medications was ceased at least 72 h before the commencement of the study and throughout the study. All volunteers were fasted overnight and at least 2 h before metronidazole formulation administration.

### 3.5. Experimental Design

Each subject was administered the two metronidazole formulations as a single oral dose of 150 mg tablet with 200 mL of water in a two-way cross-over design with a two-week wash-out period. The studied formulations were the optimized sustained release floating tablets and the conventional tablets.

### 3.6. Collection and Analysis of Blood Samples

Blood samples were obtained before (blank) and 0.5, 1.5, 2, 2.5, 3, 5, 8, 12, 14, and 24 h subsequent to metronidazole administration. The collected samples were subjected to centrifugation at 1500 rpm for 10 min. The serum obtained was frozen at −40 °C until analysis. All samples were analyzed using the established HPLC method.

### 3.7. Calculation of Pharmacokinetic Parameters

A one-compartment pharmacokinetic model with first-order absorption and elimination [24,25] was applied to the time course of metronidazole concentrations after the single oral dose in humans. Individual metronidazole serum concentration curves were utilized to estimate the metronidazole pharmacokinetic parameters. The values of peak time (T_max_) and peak height (C_max_) were obtained directly from the individual curves. The linear trapezoidal rule was employed to estimate the area under the metronidazole serum concentration–time curves (AUC_0–24_, AUC_0–__∞_). To assess the extent of absorption from the metronidazole floating tablets relative to metronidazole conventional tablets, the ratios of individual AUC_0–__∞_ values of the two formulations (i.e., relative bioavailability) were calculated. The metronidazole first-order elimination rate constant (K) and the first-order absorption rate constant (K_a_) were calculated by applying the standard equation of the one-compartment open model to individual metronidazole serum concentration–time data using WinNonlin version 2.0 pharmacokinetic software. The mean residence time of metronidazole (MRT) was calculated from the following equation:

$$\mathrm{MRT}=1/K_{a}+1/K$$

### 3.8. Statistical Analysis

Statistical comparison of the parameters determined in this study was performed by applying a one-way analysis of variance (ANOVA—single factor). Statistical significance was set at *p* < 0.05.

## 4. Results and Discussion

### 4.1. Design and Optimization of Metronidazole Floating Tablets

#### 4.1.1. Effect of Hydrogel Composition on Buoyancy

From Table 1, HPMC was the polymer of choice for this study due to its high loading capacity (50% Avicel) while maintaining its buoyancy, as shown for formulation A5. The use of HPC alone did not achieve buoyancy. The use of HMC alone resulted in floated tablets, whereas upon loading the Avicel (60 mg), the tablets sank.

#### 4.1.2. Effect of Tablet Weight and Hardness on Buoyancy

Table 3 shows the direct relationship between tablet weight and floating aspects as evaluated by measuring the maximum hardness with immediate buoyancy (MHIB). There is usually a maximum hardness range below which the tablet remains floating and after which the tablet sinks. This range is taken as a parameter to indicate the floating aspects of the tested formula regarding the direct relationship between the maximum hardness with floating features and the floating abilities. Collectively, tablets of 800 mg weight showed immediate buoyancy with maximum hardness and MHIB.

Floating tablets with different compositions were fabricated by employing the direct compression technique. The tablets must be able to gain a bulk density of less than one upon contact with the gastric fluid to achieve immediate buoyancy. Therefore, it is imperative to avoid compression forces that lead to a reduction in tablet porosity and tight adhesion of hydrocolloid particles on the tablet surface to the degree that rapid hydration is retarded. Therefore, MHIB was determined for each formulation, and it should be higher than the minimum hardness required for tablets to resist breakage during packaging and shipping. Then, this parameter is indicative of the pharmaceutical acceptance of the formulated floating tablets.

#### 4.1.3. Effect of Drug Content on Buoyancy

**Figure 2:**
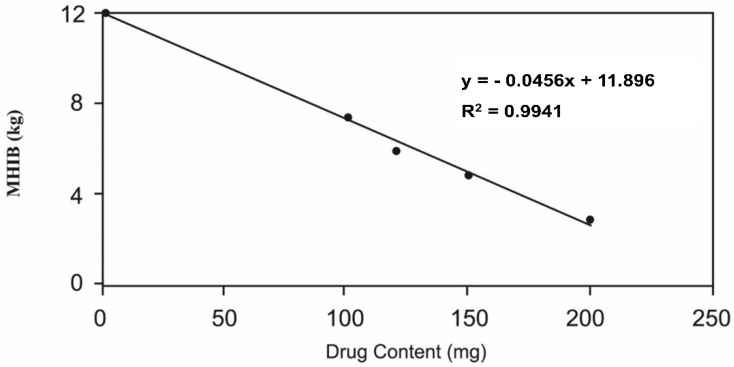
Effect of metronidazole content on the floating features of metronidazole tablets (800 mg).

Figure 2 represents the influence of metronidazole content on the floating features of the tablets. As shown in Figure 2, the maximum amount of drug that could be loaded in the HPMC matrix to prepare 800 mg floating tablets with a hardness range of 5–6 was 150 mg. Accordingly, the optimized formulation contained HPMC as the tablet matrix and 150 mg metronidazole as the active constituent. This formulation possessed a total weight of 800 mg and a pharmaceutically acceptable hardness range of 5 to 6 kg. The optimized floating tablet formulation and the conforming conventional tablets, utilized as the reference, were selected for the in vitro evaluation.

### 4.2. In Vitro Evaluation of the Tablets

The hardness, friability, content uniformity, weight variation, thickness, density, and buoyancy were evaluated.

Table 4 summarizes the values of the hardness, friability, thickness, apparent densities, floating lag time, and MHIB for the designed formulations. Tablets of all formulations complied with the USP requirements for friability, weight variation, and content uniformity (USP 2007). From Table 4, it was evident that all formulations had a hardness range of 5–6 kg and pharmaceutically accepted percentage friability values (<1.0%).

The thickness was inversely proportional to the metronidazole content. This may have been due to the decrease in polymer content, the consequent decline in tablet porosity, and the increase in apparent density. The tablets of formulations F1 to F5 with apparent densities less than 1.0 gm/cm^3^ showed immediate floating with radial and axial swelling. The tablets with apparent densities around 1.0 gm/cm^3^ (F6 and F7) were buoyant only after 35 and 42 min of floating lag time, respectively. The tablets of all formulations remained floating until completely dissolved. The density of the tablets thus correlated with the floating behavior. The principle of tablet buoyancy depends on formulating a hydrodynamically balanced system. The gel-forming hydrocolloid swells upon contact with the gastric fluid, thus maintaining the shape of the tablet and at the same time providing a bulk density of less than unity within the outer gel barrier. Tablet buoyancy is then conferred by the air trapped within the swollen polymer [26]. The buoyancy of the formulated metronidazole tablets was therefore achieved by the combination of two mechanisms: (1) increasing the tablet bulk volume when the tablet came in contact with the gastric acidic fluid as a result of hydrocolloid particle hydration and swelling, and (2) the barrier formed by the hydrocolloid particles on the surface of the tablet, which kept the internal voids in the tablet center dry.

Table 4 shows that the MHIB of formulations F1 to F5 was sufficient to provide pharmaceutically accepted tablets (more than 5 kg). Tablets of formulation F6 showed immediate floating only with a low hardness of 4 kg, whereas tablets of formulation F7 showed no immediate floating.

The results in Table 4 revealed that 150 mg metronidazole was the maximum amount that could be loaded in the 800 mg HPMC tablet (formulation F5) to give buoyant tablets with immediate floating and a pharmaceutically acceptable friability (0.686%) and hardness range (5–6 kg). Thus, formulation F5 was the optimal one based on its maximized drug content and its immediate buoyancy.

### 4.3. Density Measurements and Buoyancy Study

**Figure 3:**
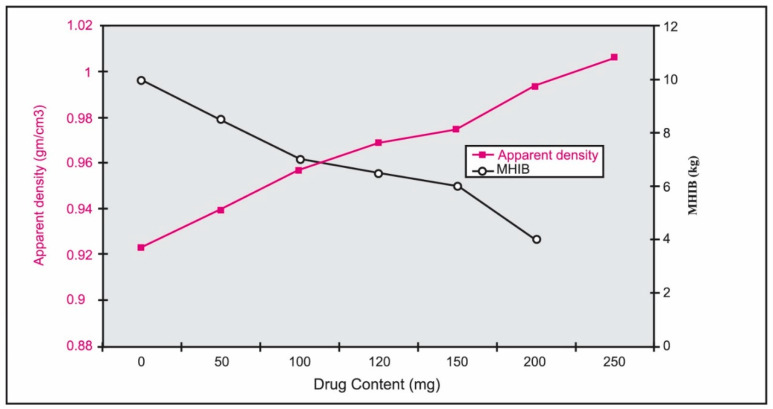
Effect of metronidazole content in 800 mg HPMC tablets on the apparent density and maximum hardness of immediate buoyancy (MHIB).

The reverse relationship between metronidazole content in HPMC tablets and the MHIB values and the direct relationship between metronidazole content and apparent density can be noticed in Figure 3. Figure 3 revealed that the increase in drug content with the concomitant decrease in the HPMC content might increase the initial density of the formulations. This may result in an increase in the apparent densities and a decrease in the MHIB values of the tablets. Indeed, two factors contributed to the tablet’s buoyancy [27]. To begin, when the hydrocolloid particles on the tablet’s surface are exposed to stomach fluids, they swell or hydrate, increasing the tablet’s bulk volume. The second is the presence of internal voids in the dried core of the tablet (porosity). These two criteria are required for the tablet to have a bulk density of less than one and float in stomach fluid. As a result, compressing these tablets to a high hardness level may limit their porosity. Furthermore, when the tablet is exposed to stomach fluids, the compressed hydrocolloid particles on the tablet’s surface are unable to hydrate rapidly, limiting the tablet’s capacity to float substantially. However, a certain degree of hardness is required for these tablets to stay buoyant.

### 4.4. In Vitro Release of Buoyant and Conventional Metronidazole Tablets

**Figure 4:**
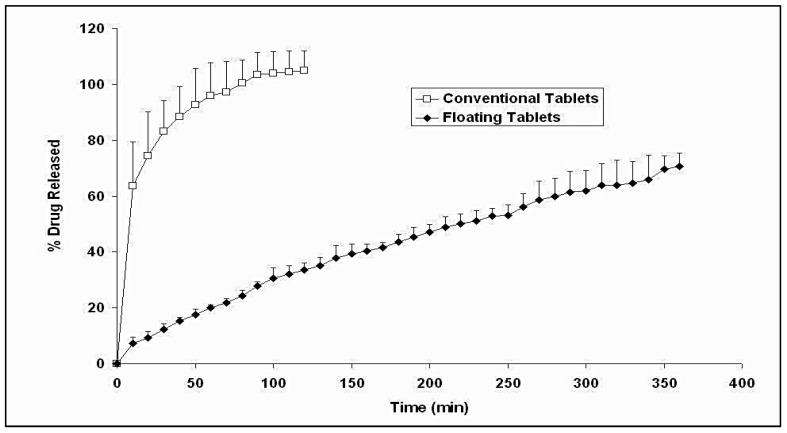
Percentage metronidazole released versus time profiles of buoyant and conventional tablets in 0.1 N HCl at 37 °C.

Figure 4 illustrates the drug release profiles from both buoyant and conventional metronidazole tablets. The conventional tablets exhibited fast release, with 100% metronidazole being dissolved in 2 h. In contrast, the floating tablets exhibited a slow and steady release rate of metronidazole over 6 h. The sustained release behavior of the metronidazole floating tablet is attributed to the interplay of different factors. The swelling of the HPMC matrix provided a gel layer that hindered the diffusion of the drug molecules toward the release medium. This phenomenon is evident in drug delivery systems composed majorly of hydrophilic polymers [28,29,30,31,32]. Another factor that is unique to the floating systems is their ability to remain on the surface of the dissolution medium for a long time, thus minimizing their contact with the surrounding aqueous medium, which reduced the surface area available for metronidazole release. Surface hydrophilization and wettability play a decisive role in releasing active pharmaceutical agents [33].

### 4.5. Kinetic Analysis of Metronidazole Release Data

The release data of the selected floating metronidazole tablets (F5) and the reference conventional metronidazole tablets were mathematically examined using the zero-order, first-order, and Higuchi diffusion models. In vitro release data of the two formulations was best fitted to the Higuchi model, where the average R^2^ values were 0.9855 and 0.9761 for the buoyant and conventional tablets, respectively.

The in vitro release rate constants (K) and release half-life values (T_1/2_) were computed from the conforming metronidazole release profiles beyond 10 min from the beginning of the release tests. The kinetic parameters with additional data are depicted in Table 5.

According to Table 5, the initial amounts of metronidazole released differ significantly depending on the tested formulation (*p* < 0.05). Conventional tablets gave a high initial drug release percentage of about 63%, whereas floating tablets showed only 7% of the drug released after 10 min. The release rate of metronidazole from the floating tablets (K = 6.278 mg min^−1/2^) was remarkably lower (*p* < 0.05) than that from the conventional tablets (K = 10.666 mg min^−1/2^). Metronidazole release from the conventional tablets was noticed to plateau after 80 min, whereas metronidazole release from the floating tablets kept going for 6 h without hitting a plateau level.

The aforementioned observations conformed to sustained release behavior with the Higuchi diffusion pattern of drug release. In this pattern, the hydrocolloid tablets convert to reservoir systems for the continuous delivery of metronidazole. When a hydrodynamically balanced dosage form interacts with an aqueous medium, hydration of the hydrocolloid commences forming a gel at the surface of the dosage form. The formed gel is responsible for controlling the rate of in-diffusion of the solvent and the out-diffusion of the drug from the dosage form. Consequently, the drug dissolves in and back diffuses with the solvent, thus generating a residing boundary within the formed gel [26]. As the gel layer on the exterior surface degrades, expansion of the adjacent hydrocolloid particles maintains the consistency of the formed gel. As a result, the gel structure constitutes a sustained-release reservoir that causes drug molecules to be slowly released by controlled diffusion across the formed gelatinous barrier.

### 4.6. HPLC Assay Validation

HPLC analysis for the determination of metronidazole in human serum has been validated in an attempt to evaluate the method in terms of linearity, selectivity, precision, and accuracy according to ICH guidelines [34].

### 4.7. Linearity

**Figure 5:**
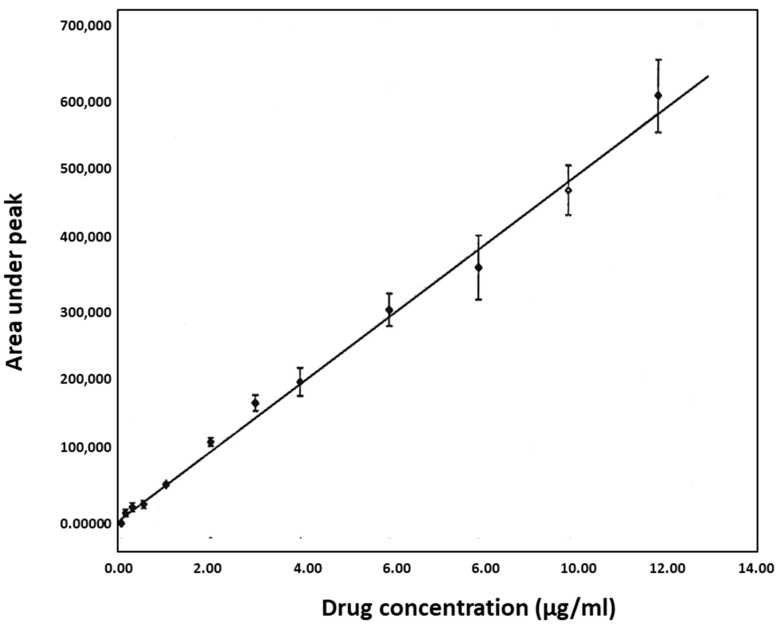
Standard curve of metronidazole (*n* = 3).

Calibration curves in the concentration range of 0.1 to 12 µg/mL were linear, as displayed in Figure 5. The equation that describes the linear relationship between the peak area (Y) and concentration (X) in the standard curves of metronidazole obtained for the entire study (*n* = 3) was:

Y=(135,572±12,037)+(58,089±3544) ·X 

### 4.8. Accuracy and Precision of the Assay

Table 6 showed that the intra-day run coefficients of variation ranged between 0.741 and 2.768%, while the inter-day run coefficients of variation ranged between 6.995 and 12.891% throughout the whole metronidazole concentration range of the calibration curve. The back-calculated metronidazole concentration of the intra-day run ranged from 96.47 to 103.592%, while those of the inter-day run ranged from 98.302 to 105.591%. These results indicated that the method used was accurate and precise.

### 4.9. Selectivity

**Figure 6:**
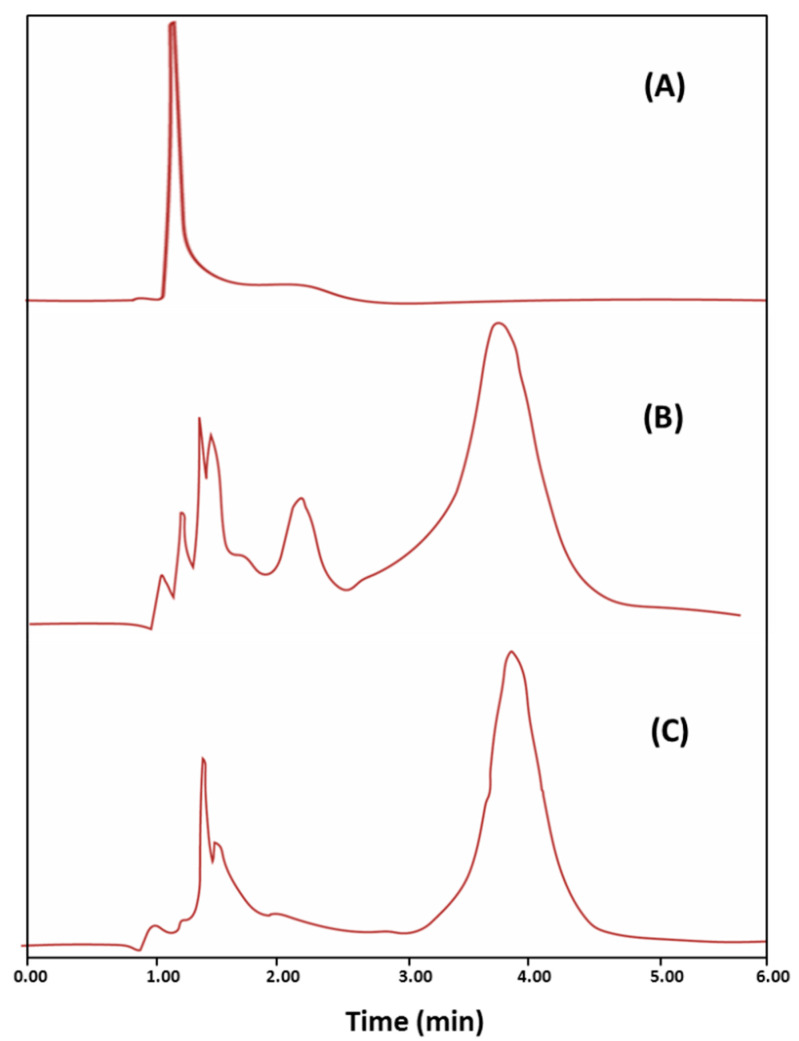
Representative HPLC chromatograms for metronidazole-free blank serum sample (**A**), serum sample spiked with metronidazole (1 µg/mL) (**B**), and serum sample obtained 6 h after metronidazole administration (**C**).

The HPLC chromatograms of a metronidazole-free blank serum sample, metronidazole-spiked serum sample, and serum sample obtained 6 h after metronidazole administration are depicted in Figure 6. The fact that the retention time of metronidazole in the standard and study samples was identical (3.2 ± 0.089) provides conclusive evidence that there were no interfering peaks for endogenous compounds that may possess the same retention time as that of metronidazole.

### 4.10. Precision of Retention Time

The precision of the retention time was assessed as the between-run percent coefficient of variation, which was very low (2.795%), indicating adequate precision. The validation parameters of the HPLC method designed for metronidazole in the present study were summarized in Table 7. In conclusion, the established HPLC method was considered valid for quantitating metronidazole in human serum.

### 4.11. Bioavailability and Pharmacokinetics

**Figure 7:**
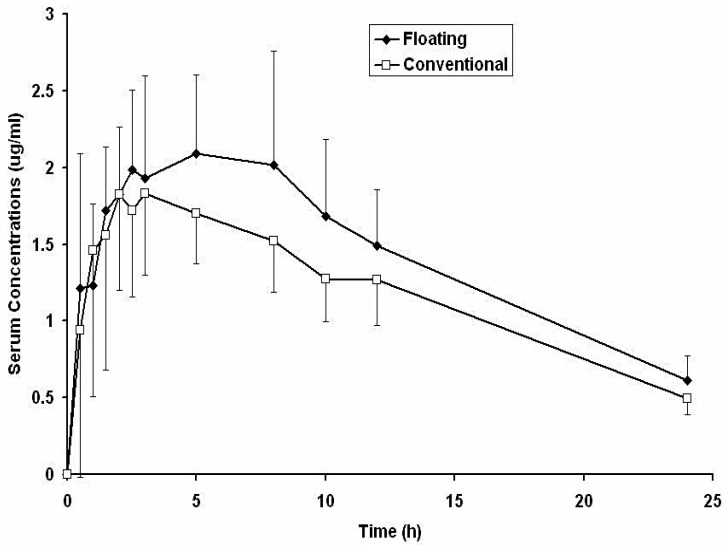
Average metronidazole serum concentration–time profiles in human volunteers following oral administration of a single dose of 150 mg as floating and conventional tablets (*n* = 6).

The average metronidazole serum drug concentration–time curves in humans following a single oral dose (150 mg) as floating and conventional tablets are shown in Figure 7. Metronidazole serum concentrations obtained with the floating tablets were insignificantly different (*p* > 0.05) during the first 8 h and significantly higher (*p* < 0.05) after 10 and 24 h as compared with the concentrations obtained with the conventional tablets. These results suggested an in vivo sustained release of metronidazole from the floating tablets.

Metronidazole pharmacokinetic parameters computed from individual curves are presented in Table 8 as mean and standard deviation values. From Table 8, it was evident that the floating tablets showed significantly higher (*p* < 0.05) T_max_, AUC_0→24_, AUC_0→__∞_, and MRT and significantly lower (*p* < 0.05) absorption rate constant (K_a_) compared with conventional tablets. No significant differences could be spotted between the C_max_, K, and T_1/2_ of the two formulations. Relative bioavailability values were computed by comparing individual AUC_0→__∞_ values for the floating tablets with those for the conventional tablets. The mean bioavailability ratio was found to be 1.223 ± 0.131. The increase in T_max_ and MRT values and the decrease in the Ka value suggested a sustained release effect for the floating tablets that conformed with the noticed in vitro release behavior of the floating tablet (Figure 4). The significantly improved bioavailability of metronidazole may have been due to both the sustained release and the gastro-retentive effects of the floating tablets.

**Figure 8:**
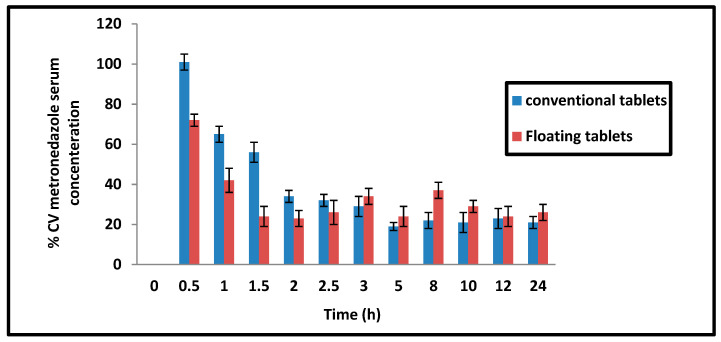
Percentage coefficient of variation of metronidazole serum concentration in conventional and floating tablets.

Metronidazole is labeled as a weak acid (pKa 2.5), which causes it to mainly be in the non-ionized absorbable form in the acidic pH of the stomach. Thus, the slow release in acidic pH achieved by the floating tablets may be very convenient for drug absorption. Sustained release dosage forms are prescribed to enhance the bioavailability of drugs with a slow and narrow absorption window [35,36]. The persistence of the floating tablet in the stomach for an extended period reduced the inter-individual variability in metronidazole serum concentrations during the first 2.5 h post-administration compared with the conventional tablets (Figure 8).

To date, several studies have investigated the potential of floating systems for metronidazole delivery. For instance, a floating drug delivery system containing metronidazole using a melt foam technique was successfully prepared [37]. In this technique, metronidazole dispersion in low-melting-point excipients (PEG 4000, labrasol, stearic acid type 50) was foamed by air under atmospheric pressure. This technique was capable of creating a low-density solid system with air-trapped voids in a micronized range. Moreover, floating microspheres composed of Eudragit E100 and polycaprolactone to enhance the gastric retention of metronidazole were prepared using an emulsification/solvent evaporation method in another study [38]. The microparticles’ performance in the simulated gastric and intestinal fluid was satisfactory. In other research, Floating Eudragit L porous beads loaded with metronidazole were fabricated using a wax removal technique [39]. It was found that increasing the wax percentage was associated with higher porosity and dissolution rate without affecting the buoyancy. Along the same line, Chitosan pH-sensitive floating hydrogels impregnated with metronidazole were slowed in the stomach of dogs for at least 48 h and demonstrated a higher effect against *H. pylori* compared with commercial metronidazole tablets [40].

A common characteristic of the floating foams, microparticles, and beads described above is that in vivo evaluation was not conducted to assess the performance in real situations. Another crucial thing is that these systems are considered complex and require sophisticated manufacturing techniques. Our formulated floating tablets comprise numerous advantages: preparation using the simple direct compression technique, lack of effervescence producing materials, and reliance on a pH-independent gel-forming agent to release metronidazole at a controlled rate regardless of the environmental pH. Therefore, the prepared tablets could be promising for enhancing both local and systemic metronidazole actions.

## 5. Conclusions

Metronidazole matrix tablets with floating and sustained release characteristics were fabricated by employing the direct compression technique utilizing the hydrocolloid HPMC. The tablets immediately floated and remain afloat until complete dissolution. The tablets showed in vitro sustained drug release according to the Higuchi model for more than 6 h in an acidic medium of 0.1 N HCl. An in vivo study in healthy volunteers revealed significantly improved bioavailability and increased mean residence time after a single oral dose of 150 mg metronidazole as floating tablets compared with conventional tablets of the same dose strength. The significant increase in the mean residence time indicated an in vivo sustained drug release. In conclusion, the prepared tablets could be promising for enhancing both local and systemic metronidazole actions.

## Figures and Tables

**Table 1:** Ingredients and additives used in the floating tablet formulations.

Formulation No.	Drug (mg)	Hydrogel (mg)			Avicel (mg)	Tablet Weight (mg)	Floating Status ^a^
		HPC	HMC	HPMC			
A1	---	600	---	---	---	600	S
A2	---	---	600	---	---	600	F
A3	---	---	---	600	---	600	F
A4	---	---	540	---	60	600	S
A5	---	---	---	300	300	600	F
A6	---	---	---	270	330	600	S

^a^ F and S denote floating and sinking statuses, respectively.

**Table 2:** Characteristics of the volunteers who participated in the single-dose pharmacokinetic study.

Subject No.	Age (Year)	Weight (kgs)
1	30	60
2	34	72
3	29	66
4	30	62
5	32	67
6	31	69
Average (±SD)	31 ± 1.788	66 ± 4.427

**Table 3:** Effect of tablet weight on floating features of tablets containing Avicel and HPMC mixture (1:1).

Tablet Weight	Hardness Range (kg)	Floating Aspects ^a^	MHIB ^b^ (kg)
400 mg	3–5	F	8
	5–7	F	
	8–9	F	
	9–10	S	
500 mg	9–10	F	9
	10–14	S	
600 mg	13–14	F	13
	14–15	S	
700 mg	13–14	F	13
	14–15	S	
800 mg	>15	F	15

^a^ F and S denote floating and sinking status, respectively. ^b^ Maximum hardness of immediate buoyancy.

**Table 4:** Composition and characteristics of the formulated floating tablets.

Formulation	Drug (mg)	Hardness (kg)	Friability (%)	Thickness (cm)	Apparent Density (gm/cm^3^)	Lag Time (min)	MHIB ^a^ (kg)
F1	0.0	5.2 ± 0.2	0.324 ± 0.01	0.563 ± 0.001	0.923 ± 0.009	0.0	10.0
F2	50	5.4 ± 0.2	0.411 ± 0.04	0.553 ± 0.004	0.939 ± 0.009	0.0	8.5
F3	100	5.3 ± 0.1	0.495 ± 0.07	0.543 ± 0.003	0.957 ± 0.010	0.0	7.0
F4	120	5.6 ± 0.3	0.573 ± 0.04	0.536 ± 0.003	0.968 ± 0.010	0.0	6.5
F5	150	5.3 ± 0.2	0.686 ± 0.06	0.533 ± 0.002	0.974 ± 0.010	0.0	6.0
F6	200	5.7 ± 0.3	0.833 ± 0.04	0.516 ± 0.005	0.994 ± 0.010	35.0 ± 5.0	4.0
F7	250	5.7 ± 0.2	0.864 ± 0.03	0.513 ± 0.004	1.01 ± 0.011	42.7 ± 2.52	-

^a^ Maximum hardness of immediate buoyancy.

**Table 5:** In vitro release kinetic parameters of metronidazole from buoyant and conventional tablets in 0.1 N HCl at 37 °C ^a^.

Formulation	Initial % Release (after 10 min)	Release Rate Constant (K) (mg min^−1/2^)	Release Half-Life (T_1/2_) (min)
Buoyant	7.293 (±2.137) ^b^	6.278 (±0.853) ^b^	181.341 (±37.905) ^b^
Conventional	63.626 (±15.802)	10.666 (±1.621)	16.321 (±5.936)

^a^ Data are given as mean ± SD (*n* = 3). ^b^ Significantly different from conventional tablets (*p* < 0.05).

**Table 6:** Precision data of the HPLC assay for metronidazole ^a^.

Nominal Concentration (µg/mL)	Found Concentration (µg/mL)	% CV	% of Nominal Concentration
Intra-day			
8	7.90 ± 0.19	2.41	98.78
10	10.35 ± 0.28	2.76	103.59
12	11.91 ± 0.32	2.72	99.29
14	13.87 ± 0.27	1.96	99.07
16	16.08 ± 0.34	2.13	100.50
18	17.36 ± 0.13	0.785	96.47
20	20.51 ± 0.15	0.741	102.55
Inter-day			
8	7.97 ± 0.81	10.19	99.64
10	10.55 ± 1.36	12.89	105.59
12	12.07 ± 1.23	10.21	100.63
14	14.15 ± 1.31	9.27	101.09
16	16.08 ± 1.12	6.99	100.53
18	17.69 ± 1.59	8.99	98.32
20	20.96 ± 1.85	8.86	104.82

^a^ Data are given as mean ± SD (*n* = 7).

**Table 7:** Validation parameters for the HPLC method for metronidazole.

Parameter	Validation Data
Retention time range	3.1–3.3 min
Linearity range	0.1–12 µg/mL
Intra-day accuracy	96.47–103.59%
Inter-day accuracy	98.32–105.59%
Intra-day precision	0.741–2.76%
Inter-day precision	6.99–12.89%

**Table 8:** Metronidazole pharmacokinetic parameters in human volunteers following administration of a single oral dose of 150 mg as floating and conventional tablets ^a^.

Parameters	Floating Tablets	Conventional Tablets
C_max_ (µg/mL)	2.490 ± 0.653	2.060 ± 0.473
T_max_ (h)	5.66 ± 2.804 ^b^	2.5 ± 2.756
K_a_ (h^−1^)	0.78 ± 0.319 ^b^	1.336 ± 0.273
K (h^−1^)	0.03 ± 0.003	0.070 ± 0.005
T_1/2_ (h)	9.52 ± 0.510	9.837 ± 0.787
AUC_0–24_ (µg·h·mL^−1^)	34.155 ± 7.099 ^b^	28.478 ± 6.394
AUC_0–∞_ (µg·h·mL^−1^)	42.658 ± 8.952 ^b^	35.535 ± 7.664
MRT (h)	15.917 ± 0.876 ^b^	14.342 ± 0.541

^a^ Data are given as mean ± SD (*n* = 6). ^b^ Significantly different from conventional tablets (*p* < 0.05). C_max_ (µg/mL): maximum serum concentration. T_max_ (h): time required to achieve maximum serum concentration. K_a_ (h^−1^): absorption rate constant. K (h^−1^): elimination rate constant. T_1/2_ (h): elimination half-life. AUC (µg·h·mL^−1^): area under the curve.

## Data Availability

Not applicable.
